# Evaluation of engineered low-lignin poplar for conversion into advanced bioproducts

**DOI:** 10.1186/s13068-022-02245-4

**Published:** 2022-12-25

**Authors:** Chien-Yuan Lin, Gina M. Geiselman, Di Liu, Harsha D. Magurudeniya, Alberto Rodriguez, Yi-Chun Chen, Venkataramana Pidatala, Faride Unda, Bashar Amer, Edward E. K. Baidoo, Shawn D. Mansfield, Blake A. Simmons, Seema Singh, Henrik V. Scheller, John M. Gladden, Aymerick Eudes

**Affiliations:** 1 DOE Joint BioEnergy Institute, Emeryville, CA 94608 USA; 2 Environmental Genomics and Systems Biology Division, Lawrence Berkeley National Laboratory, Berkeley, CA 94720 USA; 3 Department of Biomaterials and Biomanufacturing, Sandia National Laboratories, Livermore, CA 94550 USA; 4 DOE, Agile BioFoundry, Emeryville, CA 94608 USA; 5 Biological Systems and Engineering Division, Lawrence Berkeley National Laboratory, Berkeley, CA 94720 USA; 6 Department of Wood Science, University of British Columbia, Vancouver, BC Canada; 7 DOE Great Lakes Bioenergy Research Center, Wisconsin Energy Institute, Madison, WI 53726 USA; 8 Department of Bioresources and Environmental Security, Sandia National Laboratories, Livermore, CA 94550 USA; 9 Department of Plant and Microbial Biology, University of California, Berkeley, CA 94720 USA

**Keywords:** Woody biomass, *Rhodosporidium toruloides*, Ionic liquid, Saccharification, Aromatics, Fermentation

## Abstract

**Background:**

Lignocellulosic resources are promising feedstocks for the manufacture of bio-based products and bioenergy. However, the inherent recalcitrance of biomass to conversion into simple sugars currently hinders the deployment of advanced bioproducts at large scale. Lignin is a primary contributor to biomass recalcitrance as it protects cell wall polysaccharides from degradation and can inhibit hydrolytic enzymes via non-productive adsorption. Several engineering strategies have been designed to reduce lignin or modify its monomeric composition. For example, expression of bacterial 3-dehydroshikimate dehydratase (QsuB) in poplar trees resulted in a reduction in lignin due to redirection of metabolic flux toward 3,4-dihydroxybenzoate at the expense of lignin. This reduction was accompanied with remarkable changes in the pools of aromatic compounds that accumulate in the biomass.

**Results:**

The impact of these modifications on downstream biomass deconstruction and conversion into advanced bioproducts was evaluated in the current study. Using ionic liquid pretreatment followed by enzymatic saccharification, biomass from engineered trees released more glucose and xylose compared to wild-type control trees under optimum conditions. Fermentation of the resulting hydrolysates using *Rhodosporidium toruloides* strains engineered to produce *α*-bisabolene, epi-isozizaene, and fatty alcohols showed no negative impact on cell growth and yielded higher titers of bioproducts (as much as + 58%) in the case of QsuB transgenics trees.

**Conclusion:**

Our data show that low-recalcitrant poplar biomass obtained with the QsuB technology has the potential to improve the production of advanced bioproducts.

**Supplementary Information:**

The online version contains supplementary material available at 10.1186/s13068-022-02245-4.

## Background

Lignocellulosic biomass represents a renewable source of sugars and aromatics for the production of advanced bioproducts [1]. One of the advantages of second-generation bioproducts derived from biomass is their potential to mitigate greenhouse gas emissions compared to their petroleum-derived counterparts [2]. Thus, the concept of lignocellulosic biorefinery has emerged to highlight the processes that convert crops, forest materials (dedicated trees and residues), and agricultural residues into bio-based products and bioenergy [3]. One approach is the use of dedicated non-food crop feedstocks as a source of fermentable substrates for biological conversion into bioproducts and biofuels using engineered microbial strains [4]. These substrates are primarily glucose and xylose largely derived from cellulose and hemicelluloses in the plant secondary cell walls [5]. However, various challenges need to be overcome to render this approach economically viable and achieve bioproduction at relevant scales. Several strategies have been proposed, such as the engineering of crop feedstocks to facilitate their deconstruction into monomeric constituents, the use of effective solubilizing solvents for biomass pretreatment, and the cultivation of robust microbial strains tolerant to lignocellulosic inhibitors that are commonly present in biomass hydrolysates [6].

Biomass recalcitrance poses a challenge to lignocellulose deconstruction and sustainable manufacturing of advanced bioproducts [7]. It is well recognized that lignin is the primary chemical constituent that negatively affects the efficiency of polysaccharide enzymatic hydrolysis, and thereby limits the potential yields of fermentable sugars [8–10]. Several genetic engineering strategies have been proposed and implemented in crops to reduce lignin content or modify the monomeric composition toward reducing biomass recalcitrance [11, 12]. One such strategy is the in planta expression of a bacterial 3-dehydroshikimate dehydratase gene (*QsuB*) in order to reroute the endogenous pool of 3-dehydroshikimate toward production of 3,4-dihydroxybenzoate (DHB) rather than the lignin precursor phenylalanine (Additional file 1: Fig. S1) [13]. This engineering approach has been effectively applied to reduce lignin in both switchgrass and hybrid poplar [14, 15]. Hybrid poplars represent an abundant and fast-growing source of lignocellulosic biomass, but as woody species, their lignin content can exceed 25% in certain genotypes [16, 17]. The heterologous expression of QsuB in hybrid poplar successfully reduced lignin by up to 30% in woody tissues, but also resulted in the accumulation of a wide range of readily extractable metabolites that are derived from DHB [15].

The biological conversion of lignocellulosic hydrolysates requires robust microbial strains able to tolerate high concentrations of inhibitors such as monosaccharide dehydration products (e.g., furfural and 5-hydroxymethylfurfural), acetate, organic acids, and aromatics [18]. Several strains have been proposed as adequate chassis for implementing engineered metabolic pathways for conversion of lignocellulosic hydrolysates into bioproducts due to their capacity to co-metabolize biomass-derived simple sugars and aromatics. These include the bacteria *Corynebacterium glutamicum* [19, 20], *Pseudomonas putida* [21], *Sphingobium* sp. and *Novosphingobium* sp. [22], *Rhodococcus* sp. [23], and *Clostridium* sp. [24], as well several oleaginous yeast strains [25, 26]. Among the latter, the carotenogenic basidiomycete *Rhodosporidium toruloides* is an emerging platform organism amenable to metabolic engineering for upgrading lignocellulosic hydrolysates [27]. Previously, we engineered *R. toruloides* for the production of valuable bioproducts such as non-ribosomal peptides, monoterpenes, diterpenes, sesquiterpene, and fatty alcohols (FOHs) [28–33]. For example, the potential jet fuel precursors epi-isozizaene and *α*-bisabolene were produced from farnesyl pyrophosphate via the mevalonate pathway in engineered *R. toruloides* strains expressing bacterial epi-isozizaene synthase and plant-derived *α*-bisabolene synthase, respectively (Additional file 1: Fig. S2) [29, 30]. Similarly, FOHs, which are currently employed as precursors in the manufacture of detergents, cosmetics, and lubricants, accumulated in engineered *R. toruloides* strains expressing fatty acyl-CoA reductases (Additional file 1: Fig. S2) [31].

The use of ionic liquids (ILs) as eco-friendly solvents for biomass pretreatment has gained interest in the recent years because of their negligible vapor pressure, non-flammability, and good thermal and chemical stabilities. ILs are efficient at extracting lignin from biomass and have the capacity to solubilize and decrystallize cellulose during pretreatment, which facilitates subsequent enzymatic degradation [34]. Our previous work demonstrated the feasibility of growing engineered *R. toruloides* strains using wild-type poplar hydrolysates derived from IL biomass pretreatment as a carbon source [29].

The goal of the present study was to evaluate the impact of engineered low-lignin QsuB poplar on the IL-based biomass pretreatment efficiency, saccharification yields, and subsequent conversion into epi-isozizaene, *α*-bisabolene, and FOHs by engineered *R. toruloides* strains cultivated on the resulting hydrolysates.

## Results

### Poplar growth and phenotypic observations

Leaf explants of hybrid poplar (*Populus alba* × *grandidentata*; P39) from wild type (WT) and three independent QsuB transgenic lines (QsuB-1, -5, and -15) [15] were used to regenerate seedlings in vitro (Additional file 1: Fig. S3). Seedlings were propagated by plant tissue culture and transferred to soil pots in a growth chamber after root establishment. Reverse transcription-PCR (RT-PCR) was performed using mRNA isolated from the xylem tissue to confirm the presence of *QsuB* transcript in the regenerated transgenic lines (Additional file 1: Fig. S3). Consistent with Unda et al. [15], we observed noticeable phenotypic differences in wood coloration after harvesting and debarking stems, with QsuB-1 and QsuB-15 stems being darker than QsuB-5 and WT. Furthermore, cracking of the stems upon drying was apparent in the case of QsuB-1 and QsuB-15.

**Fig. 1:**
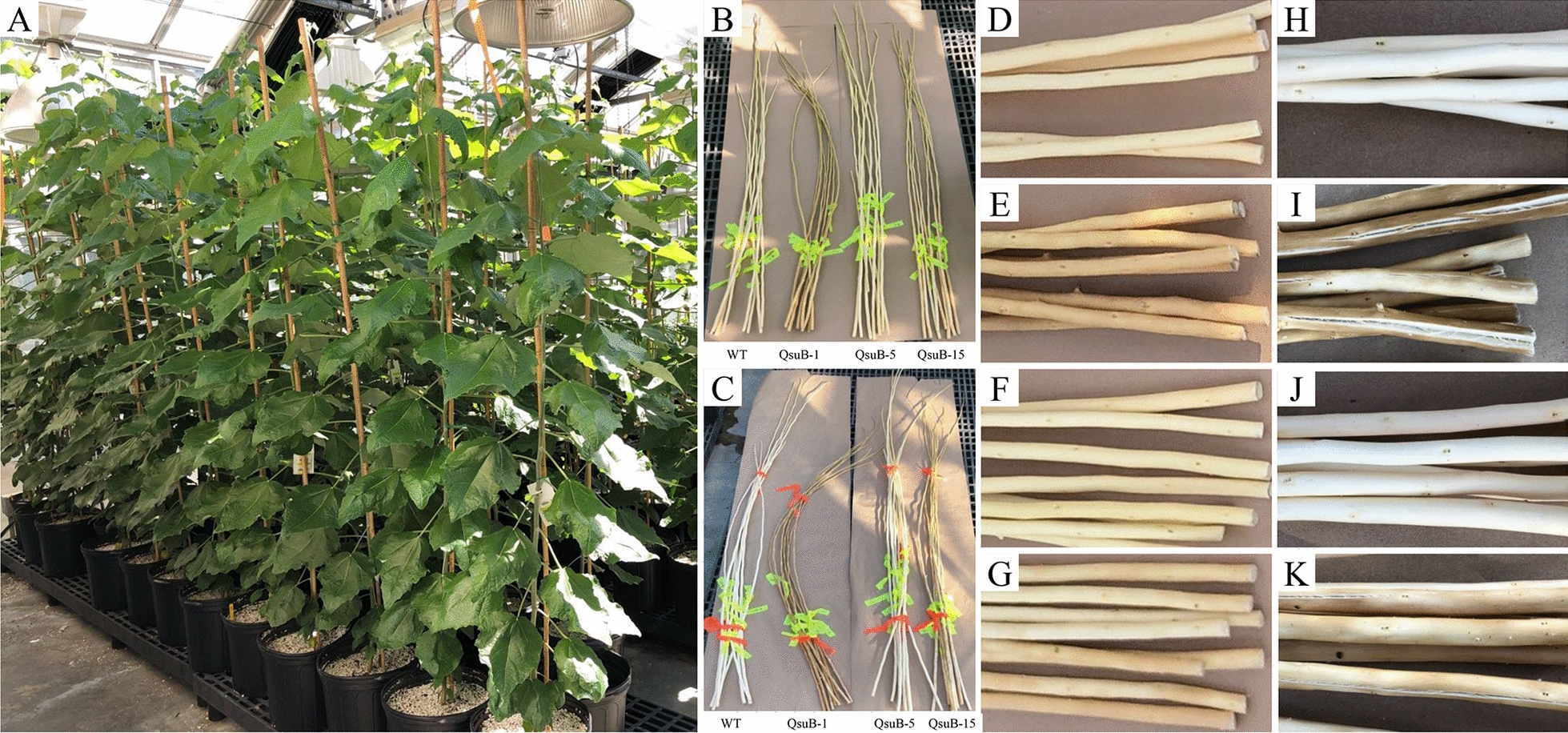
Phenotype of greenhouse-grown WT and QsuB transgenic poplar. (**A**) Plants after a 6-month growth period. (**B, D–G**) Debarked stems before drying. (**C, H–K**) Dry debarked stems. (**D, H**) WT, (**E, I**) QsuB-1, (**F, J**) QsuB-5, and (**G, K**) QsuB-15. Stems from QsuB-1 and QsuB-15 appear darker than WT and QsuB-5. Note the bending and cracking of QsuB-1 and QsuB-15 stems upon drying

**Fig. 2:**
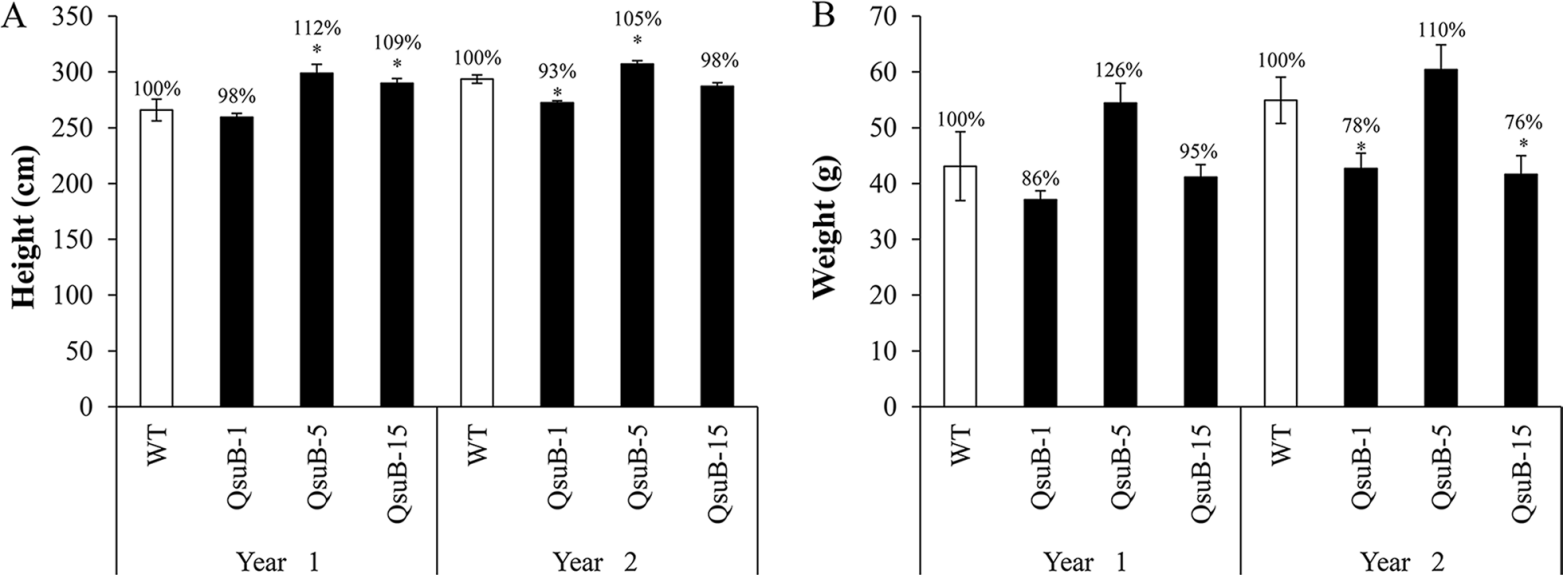
Growth parameters of WT and QsuB transgenic poplar after two successive harvests in year 1 and year 2. (**A**) Height and (**B**) dry weight of debarked stems cut 10 cm above the root collar are shown. Error bars represent the standard error of eight biological replicates. Asterisks indicate significant differences from the WT using the unpaired Student's t-test (*p < 0.05)

Seedlings were also transferred to a greenhouse for longer growth periods and two harvests over 2 years. After 6 months growth, debarked stems from transgenics showed phenotypes comparable to those previously seen in the growth chamber, consisting of various levels of wood coloration, wood shrinkage, and cracking upon drying (Fig. 1). As a measure of growth, we observed that lines QsuB-5 and QsuB-15 were ~ 12% and ~ 9% taller than WT at the first harvest, respectively, while at the second harvest lines QsuB-1 and QsuB-5 were slightly shorter and taller, respectively, than WT trees grown in the same environment (Fig. 2A). For biomass yield of the main stem, we did not observe any significant differences among transgenic lines compared to WT in the first year, however, a ~ 22–24% reduction was observed for QsuB-1 and QsuB-15 in the second year (Fig. 2B). Moreover, distinct branching patterns were noticed: QsuB-1 and QsuB-15 showed numerous sylleptic branches at the lower and middle part of the main stem, respectively, while QsuB-5 and WT did not display this phenotype.

### Biomass composition analysis of wild-type and QsuB poplar

**Fig. 3:**
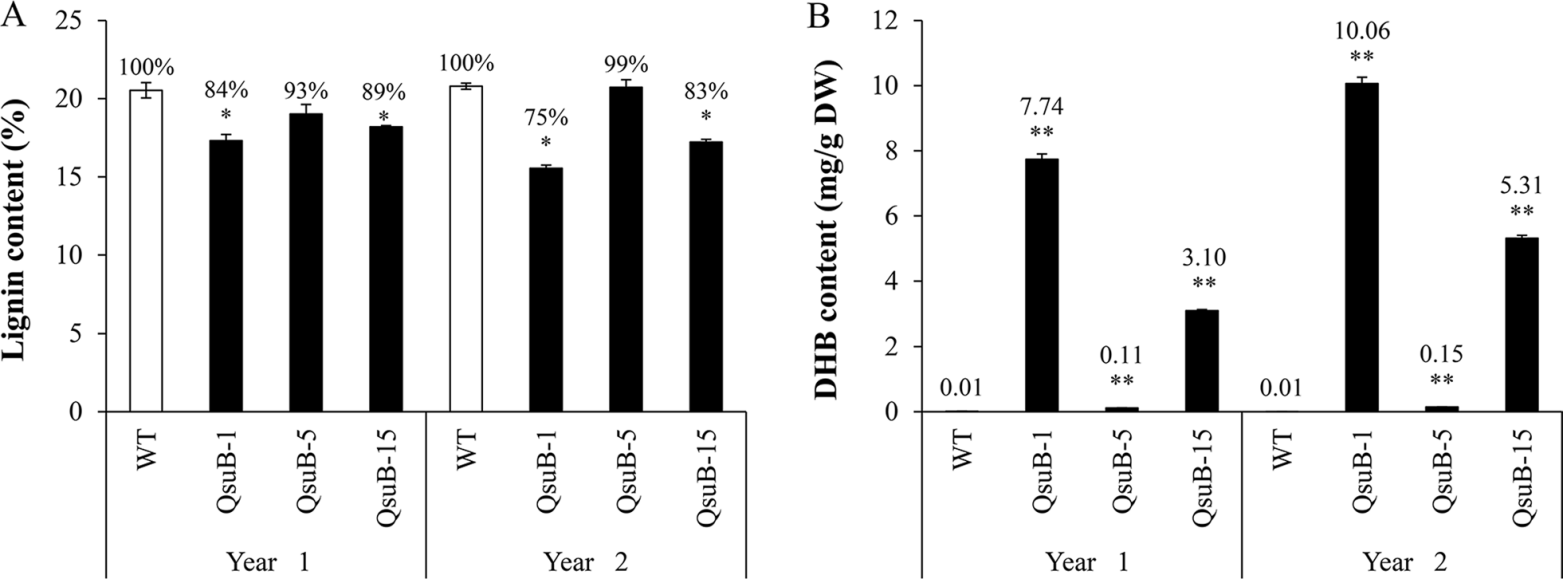
Lignin and 3,4-dihydroxybenzoate (DHB) contents in WT and QsuB poplar biomass at two different harvests. (**A**) Total lignin content expressed as a percentage (w/w) of extracted material. (**B**) DHB content in woody biomass. DW: dry weight. Error bars represent the standard error of three technical replicates conducted on a biomass pool from eight plants. Asterisks indicate significant differences from the WT using the unpaired Student's *t*-test (*p < 0.05; **p < 0.01)

For each harvest, biomass from debarked stems was subjected to cell wall and metabolite analyses. Compared to WT, lignin content was reduced by 16% (first harvest) and 25% (second harvest) in QsuB-1, and by 11% (first harvest) and 17% (second harvest) in QsuB-15, while it remained unchanged in QsuB-5 (Fig. 3A). For the two harvests, xylan content was increased by 16% and 23% in QsuB-1, and by 9% and 14% in QsuB-15, but was unchanged in QsuB-5 compared to WT (Table 1). Glucan content did not show any significant changes in the QsuB lines compared to WT (Table 1).

**Table 1:** Glucan and xylan contents in WT and QsuB poplar woody biomass

	Glucan (%)	Xylan (%)
Year 1		
WT	46.81 ± 0.10	18.54 ± 0.09
QsuB-1	45.80 ± 0.39	21.43 ± 0.08**
QsuB-5	46.94 ± 0.31	18.94 ± 0.04*
QsuB-15	46.79 ± 0.12	20.24 ± 0.02**
Year 2		
WT	46.15 ± 0.24	18.22 ± 0.24
QsuB-1	46.78 ± 0.12	22.53 ± 0.12**
QsuB-5	47.87 ± 0.11	18.54 ± 0.21
QsuB-15	46.91 ± 0.23	20.69 ± 0.15**

Values are expressed as a percentage (w/w) of extracted material

Data are means ± SE from three technical replicates conducted on a biomass pool from eight plants. Asterisks indicate significant difference from WT using the unpaired Student's *t*-test (*p < 0.05; **p < 0.01)

Next, we measured the amount of DHB in stem wood biomass, which is the product of the QsuB enzyme. Methanolic extracts from woody tissues were acid-hydrolyzed to release free DHB from its conjugated forms. For both harvests, DHB content was higher in QsuB-1 (7.7 and 10.1 mg/g dry weight (DW)) compared to QsuB-15 (3.1 and 5.3 mg/g DW), QsuB-5 (0.1 and 0.2 mg/g DW), and WT (0.01 mg/g DW) (Fig. 3B). A strong correlation was observed between the DHB content and lignin content in biomass (*R*^2^ = 0.88, Additional file 1: Fig. S4).

**Fig. 4:**
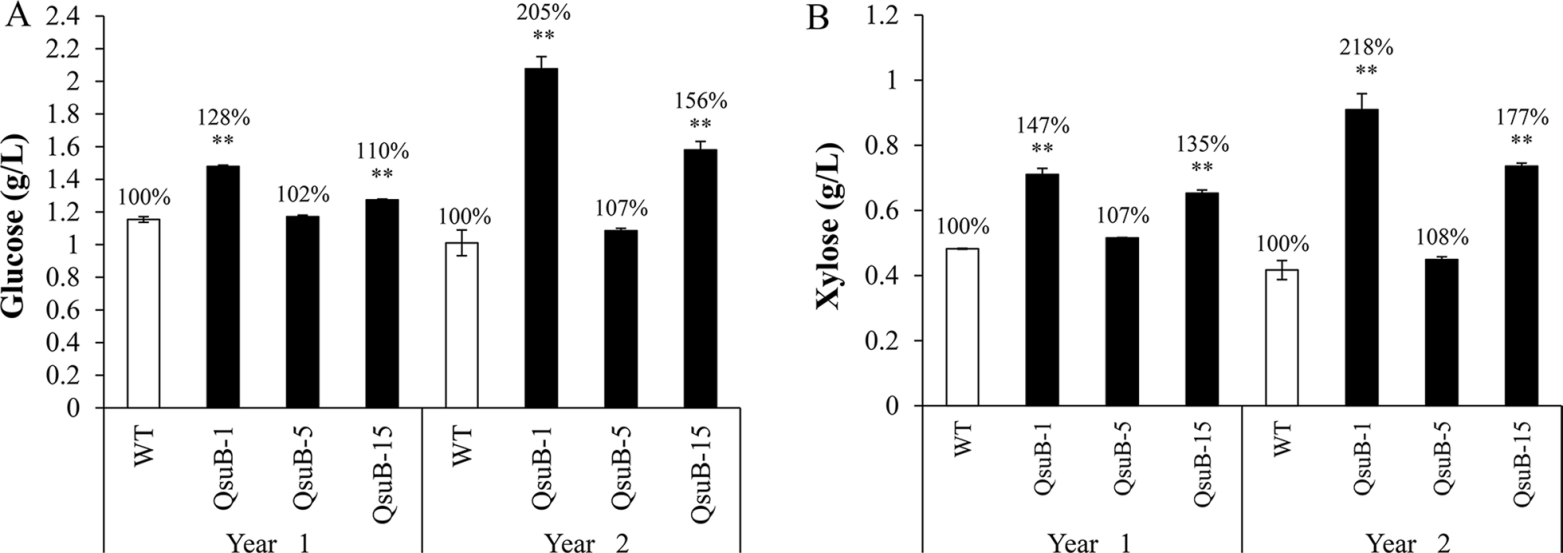
Small-scale saccharification of WT and QsuB poplar biomass at two harvests. The amount of (**A**) glucose and (**B**) xylose in hydrolysates obtained after dilute alkaline pretreatment and enzymatic hydrolysis are shown. Error bars represent the standard error of three technical replicates conducted on a biomass pool from eight plants. Asterisks indicate significant differences from the WT using the unpaired Student's *t*-test (**p < 0.01)

Finally, the recalcitrance of the QsuB lines was assessed by determining saccharification efficiency using small-scale dilute alkaline pretreatment followed by enzymatic hydrolysis. QsuB-1 and QsuB-15 released significantly higher amount of sugars compared to QsuB-5 and WT for both harvests (Fig. 4). Compared to WT, QsuB-1 showed the highest sugar titers in the hydrolysates displaying 28% and 105% increases for glucose, and 47% and 218% increases for xylose, while QsuB-15 released 10% and 56% more glucose, and 35% and 77% more xylose. Line QsuB-5 did not release more glucose nor xylose under these conditions compared to the WT for both harvests (Fig. 4).

### Conversion of poplar biomass into advanced bioproducts

Stem woody biomass obtained at the second harvest was used for conversion experiments. The ionic liquid (IL) cholinium lysinate was used as a pretreatment solvent, followed by saccharification and microbial fermentation of the hydrolysates using engineered *R. toruloides* strains for bioproduct production.

**Fig. 5:**
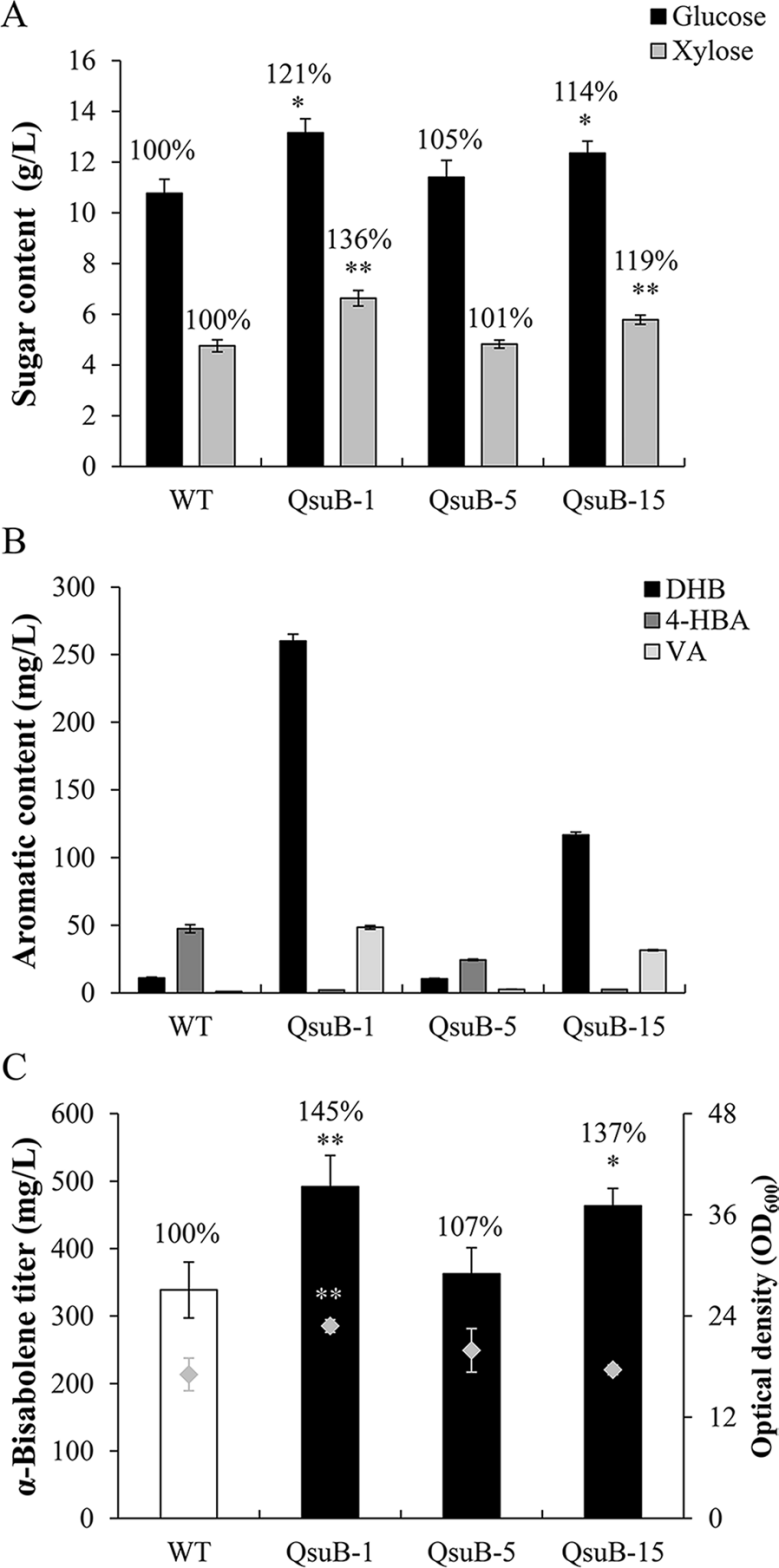
Small-scale ionic liquid (IL) pretreatment, enzymatic hydrolysis, and *α*-bisabolene production using WT and QsuB poplar biomass. The amount of (**A**) fermentable sugars and (**B**) aromatics measured in hydrolysates after IL pretreatment (105 °C for 1 h) and enzymatic hydrolysis are shown. DHB: 3,4-dihydroxybenzoate; 4-HBA: 4-hydroxybenzoate; VA: vanillate. (**C**) *α*-Bisabolene titers obtained after fermentation of hydrolysates using the *R. toruloides* GB2 strain. Grey diamonds indicate optical densities of the cultures at the end of the experiment. Error bars represent the standard error of three technical replicates conducted on a biomass pool from eight plants. Asterisks indicate significant differences from the WT hydrolysates using the unpaired Student's *t*-test (*p < 0.05; **p < 0.01)

We first used a small-scale experimental design to generate hydrolysates at low biomass loading (4% w/w). Under these conditions, hydrolysates from QsuB-1 showed 21% more glucose and 36% more xylose compared to WT, while those from QsuB-15 had 14% and 19% more glucose and xylose, respectively. Sugar titers in QsuB-5 hydrolysates were similar to those measured in WT hydrolysates (Fig. 5A). Next, measurement of aromatics in hydrolysates showed high DHB titers for QsuB-1 (~ 260 mg/L) and QsuB-15 (~ 120 mg/L), which represents 26- and 12-fold increases, respectively, compared to hydrolysates from both QsuB-5 and WT (Fig. 5B). In addition, hydrolysates from the QsuB lines had lower amount of 4-hydroxybenzoic acid (4-HBA) and higher amount of vanillic acid (VA) compared to WT hydrolysates which contained ~ 50 mg/L of 4-HBA and 1 mg/L of VA (Fig. 5B). After pH adjustment, hydrolysates were inoculated with the engineered *R. toruloides* GB2 strain for *α*-bisabolene production. After 1 week of cultivation, compared to the titer achieved using hydrolysates from WT biomass, the production of *α*-bisabolene was 45% and 37% higher in the case of hydrolysates derived from QsuB-1 and QsuB-15 biomass, respectively. *α*-Bisabolene production achieved with QsuB-5 hydrolysates was not significantly different from that obtained with WT hydrolysates (Fig. 5C). At the end of the fermentation, higher yeast cell densities were observed with QsuB-1 hydrolysates (Fig. 5C). Overall, these small-scale conversion assays validated the use of QsuB poplar biomass in combination with *R. toruloides* for bioproduct production.

**Fig. 6:**
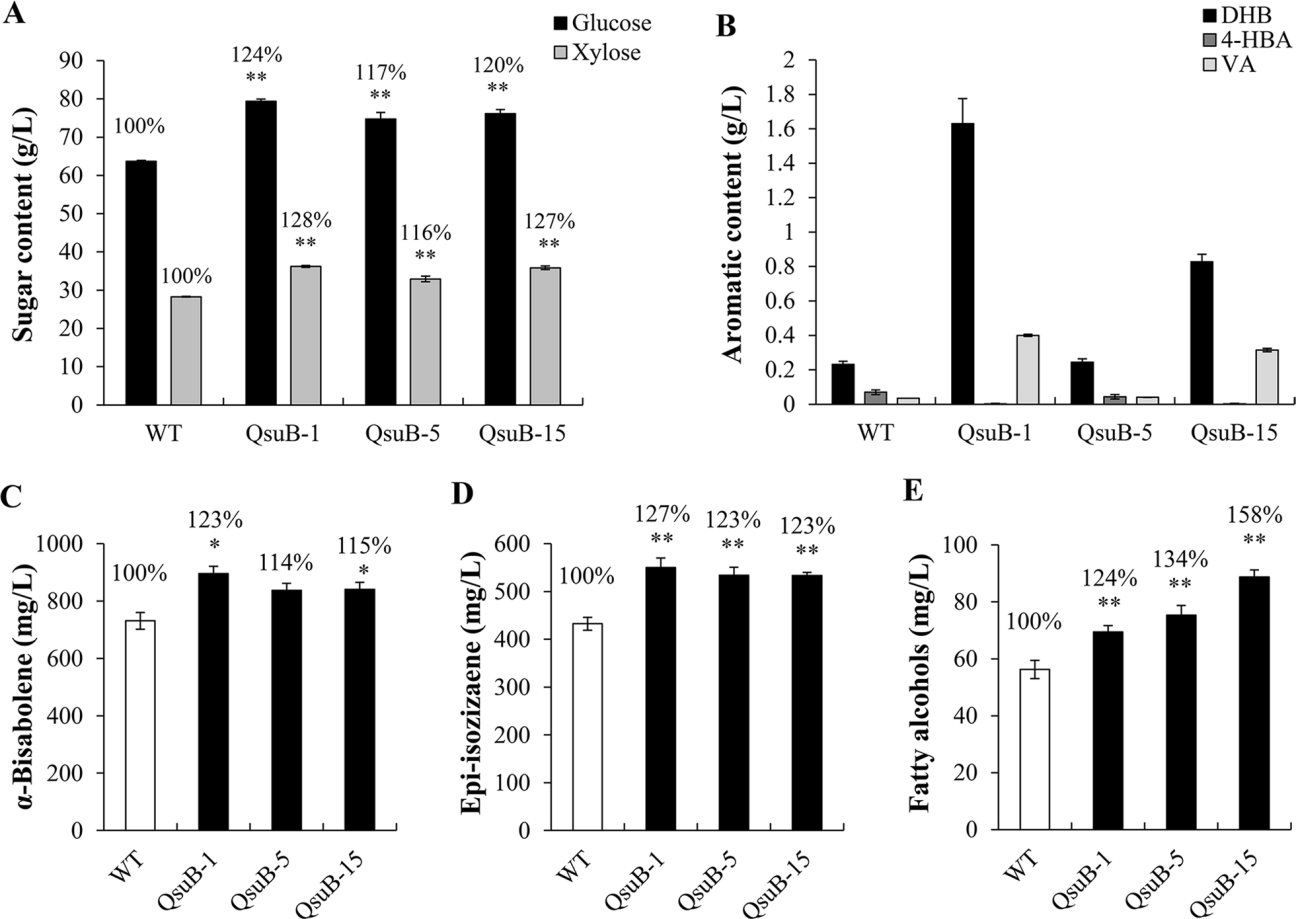
Large-scale IL pretreatment, enzymatic hydrolysis, and bioproduct production using WT and QsuB poplar biomass. (**A**) Amount of glucose and xylose in hydrolysates after IL pretreatment (140 °C for 3 h) and enzymatic hydrolysis. (**B**) Aromatic content in hydrolysates. Titers of (**C**) *α*-bisabolene, (**D**) epi-isozizaene, and (**E**) fatty alcohols obtained after fermentation of the hydrolysates using the *R. toruloides* strains GB2, EIZS2, and maquFOH are shown. Error bars represent the standard error of three biological replicates from independent yeast cultures. Asterisks indicate significant differences from the WT hydrolysate using the unpaired Student's *t*-test (*p < 0.05; **p < 0.01)

Next, new hydrolysates were generated at a larger scale, with higher biomass loading (20% w/w) and harsher pretreatment conditions (140 °C for 3 h) using a 1-L benchtop reactor. Elevated amounts of glucose (+17–24%) and xylose (+16–28%) were measured in hydrolysates obtained from the biomass of engineered lines compared to WT (Fig. 6A). DHB titers were ~ 0.25 g/L in hydrolysates from QsuB-5 and WT, and reached ~ 1.6 g/L and ~ 0.8 g/L in QsuB-1 and QsuB-15 hydrolysates, respectively (Fig. 6B). 4-HBA and VA contents were, respectively, reduced and increased in QsuB hydrolysates compared to WT (Fig. 6B). Hydrolysates were subjected to fermentation for the production of *α*-bisabolene, epi-isozizaene, and fatty alcohols (FOHs) using three different engineered *R. toruloides* strains. The *α*-bisabolene titer was 730 mg/L after cultivation of the *R. toruloides* GB2 strain using the WT hydrolysate as the carbon source, and it significantly increased by 23% and 15% when the hydrolysates derived from QsuB-1 and QsuB-15 biomass were used, respectively (Fig. 6C). Epi-isozizaene production was achieved using the *R. toruloides* EIZS2 strain. An epi-isozizaene titer of 430 mg/L was achieved when this strain was grown on the WT hydrolysate, which increased by 23–27% in the case of hydrolysates derived from the QsuB1 and QsuB15 engineered lines (Fig. 6D). Fermentation was conducted using the *R. toruloides* maquFOH strain for the production of FOHs. Compared to the FOH titer obtained with the WT hydrolysate (~ 55 mg/L), FOH production was enhanced by 24%, 34%, and 58% using QsuB-1, QsuB-5, and QsuB-15 hydrolysates, respectively (Fig. 6E).

## Discussion

We show in this work that using low-recalcitrant poplar generated via the insertion of the QsuB gene has a positive impact on the yield of monomeric aromatics and sugars released during biomass pretreatment and saccharification, which in turn leads to higher titers of bioproducts after fermentation of the hydrolysates with engineered *R. toruloides* strains. The higher amount of sugars and aromatics in the hydrolysates from QsuB biomass has marginal effect on the final yeast cell density compared to cultures grown on hydrolysates from WT biomass (Additional file 1: Fig. S5), suggesting that these substrates are somehow directed towards bioproduct production. The occurrence of an intradiol 3,4-cleavage pathway that converts DHB into the tricarboxylic acid cycle intermediates succinyl-CoA and acetyl-CoA has been recently proposed in *R. toruloides* [35]. Therefore, since *α*-bisabolene, epi-isozizaene, and FOHs are produced from acetyl-CoA via the mevalonate and fatty acid pathways in engineered *R. toruloides* (Additional file 1: Fig. S2), it is likely that DHB accumulated in QsuB poplar hydrolysates was converted into these three target bioproducts during yeast cultivation.

Consistent with the known enzymatic activity of QsuB, analysis of the composition of QsuB poplar biomass indicates a perturbation of the metabolic flux toward phenylalanine biosynthesis, hence the negative correlation observed between DHB content and overall lignin content among the QsuB poplar trees (Additional file 1: Fig. S4). DHB was previously shown to accumulate as conjugated glycoside ester and phenolic glycoside forms in the QsuB lines [15], nevertheless a significant amount of free DHB was detected in hydrolysates, which indicates that the IL-based pretreatment and saccharification conditions are effective at hydrolyzing these conjugates (Fig. 6B). Other aromatics were also detected in hydrolysates: higher amount of VA in the case of the QsuB lines suggests that a portion of the DHB is methylated by endogenous poplar *O*-methyltransferase(s), while reduction of 4-HBA indicates that QsuB activity competes with the natural formation of 4-HBA ester groups on lignin. Higher sugar yields are obtained after saccharification of QsuB poplar biomass likely as a result of lower lignin content, which validates the role of lignin as an important contributor to biomass recalcitrance. In addition, the relative increase of *p*-hydroxyphenyl units and concomitant reduction in the degree of polymerization of the ensuing lignin presumably reduce lignin recalcitrance in QsuB poplar [15]. Both glucose and xylose titers were higher in QsuB poplar hydrolysates compared to WT, implying that both cellulose and hemicellulose (i.e., xylans) were more accessible to cell wall degrading enzymes, in addition to the fact that xylose content is much higher in QsuB woody biomass. In contrast, lignin content was not reduced in QsuB-5 line compared to WT control, but this line had increased sugar yields after saccharification following more drastic pretreatment conditions (Fig. 6A). These observations could be related to the incorporation of DHB in lignin and the formation of more labile benzodioxane structures (i.e., the so-called zip-lignin) within the polymer in QsuB poplar [15]. These structures may be prone to cleavage under the current pretreatment conditions and lead to reduced lignin recalcitrance despite similar lignin content in line QsuB-5 compared to WT. More detailed analyses of lignin recovered in hydrolysates from QsuB-5 biomass, such as its molecular weight, polydispersity index, and interunit linkages are warranted to fully understand the exact mechanisms involved in this finding. Moreover, understanding the structural basis of the wood phenotypic abnormalities observed in lines QsuB-1 and QsuB-15 would be informative for future engineering toward mitigating biomass yield penalty. For example, the low-lignin QsuB trait could be stacked with traits that enhance wood density and mechanical resistance [36, 37], as well as other beneficial traits previously introduced to trees via plant biotechnology [38, 39].

Hydrolysates from QsuB poplar biomass generated after IL pretreatment and saccharification in a 1-L bioreactor enabled higher titers of *α*-bisabolene, epi-isozizaene, and FOHs after fermentation. Further work needs to be done to evaluate the efficiency of biomass saccharification and the performance of the engineered yeast strains at a larger scale. Although we previously successfully cultivated a *R. toruloides α*-bisabolene-producing strain on sorghum hydrolysates produced in 2-L and 20-L bioreactors [40], the strains GB2, EIZS2, and maquFOH used in the current study have never been tested at those scales or using poplar-derived biomass as a feedstock.

Several valuable chemicals such as muconate, 2-pyrone-4,6-dicarboxylate, 2,5-pyridinedicarboxylate, or *β*-ketoadipate have been produced using DHB as precursor in engineered microbial strains [41]. Therefore, biomass from QsuB poplar represents an appealing feedstock for biological conversion into these other bioproducts considering the substantial DHB titers measured in hydrolysates from QsuB poplar. In this regard, we previously demonstrated in Arabidopsis that co-expression of QsuB with a feedback-insensitive 3-deoxy-D-arabinoheptulosonate 7-phosphate synthase from *Escherichia coli* resulted in a ~ 250% increase in DHB content in biomass (Additional file 1: Fig. S1) [42]. Thus, a similar genetic modification to QsuB poplar could be employed to further enhance DHB accumulation in wood and increase DHB titers in biomass hydrolysates.

Finally, QsuB poplar will need to be grown in the field to determine growth performance, stress resilience, susceptibility to microbial colonization and degradation, and biomass yield across multiple harvests under natural conditions. Line QsuB-5 represents a good candidate for further evaluation considering that it does not show any growth phenotype nor yield penalty under greenhouse conditions, but still released more sugars after saccharification and enabled higher titers of bioproducts after fermentation.

## Conclusions

We demonstrate the suitability of the QsuB lignin engineering strategy to improve the production of advanced jet fuel blendstocks and fatty alcohol-derived bioproducts. Using an integrated process that combines engineered QsuB poplar biomass, one-pot IL-based pretreatment and saccharification, and engineered *R. toruloides* yeast strains, we obtained higher yields of fermentable substrates in hydrolysates derived from modified poplar, which enabled higher titers of advanced bioproducts after fermentation. The characteristics of alternative dedicated bioenergy crops (e.g., switchgrass, sorghum, etc.) modified with QsuB and the impact of QsuB-modified biomass on the performance of different engineered microbial strains toward production of a wider range of bio-based products will need to be addressed.

## Materials and methods

### Poplar growth conditions

Hybrid poplar (*Populus alba* × *grandidentata*; p39) wild type (WT) and QsuB transgenic lines were generated as previously described [15] and shipped to the Joint BioEnergy Institute (Emeryville, CA, USA) under an APHIS import permit (#18-128-103n). Seedlings were clonally propagated and maintained in a modified rooting medium (RM) containing 1-naphthaleneacetic acid (0.01 μM) and timentin (200 mg/L) instead of indole-3-butyric acid and cefotaxime [43]. Murashige and Skoog salt mixture and vitamins were purchased from PhytoTechnology Laboratories (Lenexa, KS). After several rounds of propagation, seedlings were transferred to soil (Sunshine^®^ Advanced Mix #4 Growing Mix [Sun Gro Horticulture, Agawam, MA]) and grown in a growth chamber (22 °C, light intensity of 180 µmoles m^−2^ s^−1^, 70% humidity), and eventually transferred to the greenhouse (minimum temperature set at 22 °C). For harvest, stems were cut 10 cm above the root collar, the phloem was peeled from the xylem, and the woody biomass was air-dried prior further processing. Dry debarked stems were ground into powder using a Wiley mill equipped with a 2-mm sieve (Thomas Scientific, Swedesboro, NJ). The biomass from eight trees per genotype was pooled for composition analyses and conversion into bioproducts.

### Reverse transcription-PCR (RT-PCR)

RNA extraction from 2-month-old xylem tissue and cDNA synthesis was performed following the method described in Lin et al. [45]. Primer sequences specific to *QsuB* and to the reference gene *PtTIF5A* are listed in Additional file 1: Table S1.

### DHB extraction and quantification

Metabolites were extracted from dry wood powder using 80% (v/v) methanol–water as solvent as previously described [13]. For each sample, 30 mg of biomass was sequentially extracted four times with 1 mL of solvent at 70 °C. The 4-mL extracts were mixed with 2 mL of HPLC grade water and cleared by centrifugation at 3000×*g* for 5 min. After centrifugation, extracts were filtered through Amicon Ultra centrifugal filters (3 kDa MW cut-off, EMD Millipore, Billerica, MA) at 12,000×*g* for 60 min at 4 °C. For DHB quantification, a 500 μL aliquot of the filtered extracts was dried under vacuum and hydrolyzed with 1 M HCl for 3 h at 90 °C to release the DHB aglycone form, followed by three ethyl acetate partitioning steps as previously described [13]. DHB was analyzed using high-performance liquid chromatography–electrospray ionization time-of-flight-mass spectrometry (HPLC-ESI TOF–MS) as previously described [44]. Quantification was performed via 6-point calibration curves of a standard compound (Sigma-Aldrich, MO, USA). The monoisotopic *m/z* (negative ionization) of deprotonated DHB is 153.01933.

### Biomass composition analysis

Stem wood powder was sequentially extracted with 80% ethanol (twice), acetone, chloroform/methanol (1:1), and acetone. The Klason method was used to determine total lignin (acid-soluble and acid-insoluble), glucose, and xylose following the procedure described in Lin et al. [45]. Glucose and xylose were quantified by HPLC using the method described in Lin et al. [42].

### Dilute alkaline pretreatment and saccharification

Biomass pretreatment (15-mg scale) using 62.5 mM sodium hydroxide followed by enzymatic polysaccharide hydrolysis and measurement of released glucose and xylose was performed using high-performance anion-exchange chromatography following the method described in Scavuzzo-Duggan et al. [46].

### Preparation of the ionic liquid (IL) cholinium lysinate

Cholinium lysinate ([Ch][Lys]) was synthesized by dissolving lysine monohydrate (0.4 mol, 65.68 g) in 100 mL deionized water at room temperature to obtain a clear solution (light lime-yellow). The flask was then mounted on an ice-bath (3–5 °C), and N_2_ was purged for 20–30 min. Subsequently, 46 wt% of choline hydroxide in water (0.4 mol, 105.15 g) was added dropwise to the lysine solution while maintaining the temperature at 3–5 °C. The mixture was stirred for 48 h at room temperature. Excess water was removed under reduced pressure, and the mixture was added to acetonitrile/methanol (9:1, v/v) to remove the excess starting materials. Finally, the solvents were removed under reduced pressure, and the mixture was freeze-dried to obtain the final product (yield ~ 95%, light orange).

### IL pretreatment and saccharification

For small-scale IL pretreatment, 80 mg of dry woody biomass was mixed thoroughly with 440 µL of 10% IL in a screw cap tube, and then heated at 105 °C for 1 h. After cooling, 1560 µL of water was added, vortexed until the biomass pellet was dissolved, and the pH of the biomass solution was adjusted to 5 using 10 M HCl. Subsequently, 30 mg protein/g of biomass of commercial enzyme mixtures, Cellic CTec3 and HTec3 (9:1 v/v), were added to the biomass slurry to carry out enzymatic hydrolysis (saccharification) at 50 °C for 72 h. After enzymatic hydrolysis, the hydrolysate was adjusted to pH 6 using 6 M NaOH. The samples were filtered using 0.45-µm funnel filters followed by 0.2 µm to remove the solid residual to get the final hydrolysate solution.

The procedure described in Sundstrom et al. [40] was used for large-scale one-pot IL pretreatment and saccharification. A 1-L 4520 Parr bench top reactor (Parr Instrument Company, Moline, IL, USA) equipped with three-arm, self-centering anchor with PTFE wiper blades was employed for all reactions. The pretreatment vessel was loaded with 25% w/w biomass and an 80% w/w aqueous fraction consisting of 90% deionized water and 10% [Ch][Lys]. During pretreatment, the reaction vessel was heated to a temperature of 140 °C for 3 h under constant mixing. After cooling the reactor, the pH of the mixture was adjusted to 5 using 50% (v/v) sulfuric acid and saccharification was conducted by adding the Cellic CTec3 and HTec3 enzyme mixtures as described above. The reaction vessel was mixed and heated to 50 °C for 72 h. The resulting hydrolysates were filtered prior to metabolite analysis and fermentation experiments. Monosaccharides and aromatics were quantified by HPLC and liquid chromatography mass spectrometry (LC–MS) analysis as previously described [42].

### *α*-Bisabolene and epi-isozizaene production

The *R. toruloides* strains *EIZS2* (JBEI registry ID: JPUB_013532) [29] and *GB2* (ABF registry ID: ABFPUB_000311) [30] were used to produce the sesquiterpenes epi-isozizaene and (*E*)-α-bisabolene (*α*-bisabolene), respectively. Strains were grown on YPD (10 g/L yeast extract, 20 g/L peptone, and 20 g/L glucose) agar plates at 30 °C for 3 days. Single colonies were inoculated into culture tubes containing 10 mL YPD, 100 μg/mL carbenicillin, and 100 μg/mL cefotaxime and cultured overnight at 30 °C with shaking at 200 rpm. Cells were collected by centrifugation (3000×*g* for 5 min) and washed in sterile water. Cells were resuspended in sterile water and used to inoculate a culture medium consisting of 50% (v/v) YPD and 50% (v/v) poplar hydrolysate supplemented with 5 g/L ammonium sulfate, 100 μg/mL carbenicillin, and 100 μg/mL cefotaxime in a final volume of 2.5 mL and at a starting optical density OD_600_ = 1_._ After overnight growth, cells were collected by centrifugation, washed in sterile water, and used to inoculate 0.8 mL of a culture medium consisting of the poplar hydrolysate supplemented with 5 g/L ammonium sulfate, 100 μg/mL carbenicillin, and 100 μg/mL cefotaxime. Cultures were started at OD_600_ = 0.5 and grown for 7 days in 48-well flower plates with gas-permeable sealing film inside a Multitron incubation shaker at 950 rpm, 30 °C, and 70% humidity. A 20% (v/v) dodecane overlay was used with an internal standard of 250 mg/L pentadecane.

### Fatty alcohols (FOHs) production

The *R. toruloides* maquFOH strain (JBEI registry ID: JPUB_013267) [31] was used for the production of FOHs. Cells from a single colony were grown overnight in liquid YPD medium at 200 rpm and 30 °C. Overnight cultures were used to inoculate a culture medium (starting OD_600_ = 0.1) containing 50% (v/v) poplar hydrolysate as carbon source, 100 mM phosphate solution buffered at pH 6.0, 10 μM FeSO_4_, 1 g/L ammonium sulfate, 0.1% tergitol, and a 20% (v/v) dodecane overlay. The cells were cultured for 5 days at 990 rpm, 70% humidity, and 30 °C.

### Bioproduct measurements

To quantify epi-isozizaene and *α*-bisabolene, dodecane was used to make dilutions (1:50 and 1:33, respectively) of the overlays in a total volume of 100 μL. Hexadecane (40 mg/L) was used as an internal standard during dilution, as well as caryophyllene (40 mg/L) in the case of epi-isozizaene samples. *α*-Bisabolene standards were used for quantification. Epi-isozizaene titer was determined using a conversion factor calculated by comparing SIM and SCAN corrected peak area of *α*-bisabolene to that of epi-isozizaene. The diluted overlay samples were analyzed by gas chromatography–mass spectrometry (GC–MS) using an Agilent 6890 Plus gas chromatograph, operating with an Agilent 5973 Network mass spectrometer. 1 μL of each sample was injected by a CombiPal autosampler. Analytes were separated on a DB-5MS column using the following oven parameters: hold for 0.75 min at an initial temperature of 100 °C, followed by a temperature ramp of 40 °C/min to 300 °C. The mass spectrometer was operated in selected ion mode, with target ions (*m/z*) of 71, 85, 119, 161, 189, and 204. Analysis was performed on Enhanced ChemStation.

To quantify FOHs, 100 μL of 1-tridecanol C13:0 (1 g/L in dodecane) was added to the cultures as internal standard. Then 30 μL of the dodecane overlay was sampled and diluted into 300 μL ethyl acetate. FOHs were quantified by gas chromatography-flame ionization detector (GC-FID) using a method previously described [47] and the following temperature program: the column was equilibrated at 150 °C for 3 min, followed by a ramp to 245 °C at 20 °C/min, and held at this temperature for 6 min. Final FOHs concentrations were determined by comparing the peak areas of cetyl alcohol C16:0, palmitoleyl alcohol C16:1, 1-heptadecanol C17:0, stearyl alcohol C18:0, and oleyl alcohol C18:1 to that of the C13:0 internal standard with calibration curves from a custom FOHs standard mix prepared in ethyl acetate.

All titers are normalized to the aqueous volume of the fermentation.

## Supplementary Information

**Additional file 1: Table S1.** Primers used for RT-PCR. **Figure S1.** The QsuB engineering strategy. **Figure S2.** Metabolic routes for microbial conversion of fermentable sugars from lignocellulosic hydrolysates into sesquiterpenes (epi-isozizaene and *α*-bisabolene) and fatty alcohols. **Figure S3.** Propagation and characterization of WT and QsuB poplar lines. **Figure S4.** Correlation between lignin content and DHB content in WT and QsuB biomass. **Figure S5.** Optical density of *R. toruloides* cultures after cultivation on large-scale poplar hydrolysates.

## Data Availability

All the data supporting the conclusions of this article are included within the article and its additional file.

## Abbreviations

DW: Dry weight
3-DHS: 3-Dehydroshikimate
DHB: 3,4-Dihydroxybenzoate
IL: Ionic liquid
4-HBA: 4-Hydroxybenzoate
VA: Vanillate
FOHs: Fatty alcohols
