# Supplementary material for: Evaluation of engineered low-lignin poplar for conversion into advanced bioproducts
Source: Biotechnol Biofuels Bioprod. 2022 Dec 25;15:145. doi: 10.1186/s13068-022-02245-4 (PMC9790118; doi:10.1186/s13068-022-02245-4)
Supplement: Supplementary file 1 — Additional file 1: Table S1. Primers used for RT-PCR. Figure S1. The QsuB engineering strategy. Figure S2. Metabolic routes for microbial conversion of fermentable sugars from lignocellulosic hydrolysates into sesquiterpenes (epi-isozizaene and α-bisabolene) and fatty alcohols. Figure S3. Propagation and characterization of WT and QsuB poplar lines. Figure S4. Correlation between lignin content and DHB content in WT and QsuB biomass. Figure S5. Optical density of R. toruloides cultures after cultivation on large-scale poplar hydrolysates. [file 13068_2022_2245_MOESM1_ESM.pdf]

Evaluation of engineered low-lignin poplar for conversion into advanced bioproducts

Chien-Yuan Lin, Gina M. Geiselman, Di Liu, Harsha D. Magurudeniya, Alberto Rodriguez, Yi-Chun Chen, Venkataramana Pidatala, Faride Unda, Bashar Amer, Edward E. K. Baidoo, Shawn D. Mansfield, Blake A. Simmons, Seema Singh, Henrik V. Scheller, John M. Gladden, Aymerick Eudes

Additional File 1

Table S1. Primers used for RT-PCR
Figure S1. The QsuB plant engineering strategy.
Figure S2. Metabolic routes for microbial conversion of fermentable sugars from lignocellulosic hydrolysates into sesquiterpenes (epi-isozizaene and α-bisabolene) and fatty alcohols.
Figure S3. Propagation and characterization of WT and QsuB poplar lines.
Figure S4. Correlation between lignin content and DHB content in WT and QsuB poplar.
Figure S5. Optical density of *R. toruloides* cultures after cultivation on large-scale poplar hydrolysates.

Table S1. Primers used for RT-PCR

Primer name	Sequence (5' to 3')	Target gene
QsuB_qRT-F	ACATGGTGGCTCCGTTCACC	3-Dehydroshikimate
QsuB_qRT-R	GTTGCTGGGAAGGGTGGAGA	Dehydratase (*QsuB*)
PtTIF5A_qRT_F	GGCATTAAGTTTTGTCGGTCTG	Translation Initiation
PtTIF5A_qRT_R	GCGGTTCATCATTTCATCTGG	Factor 5A (*PtTIF5A*)

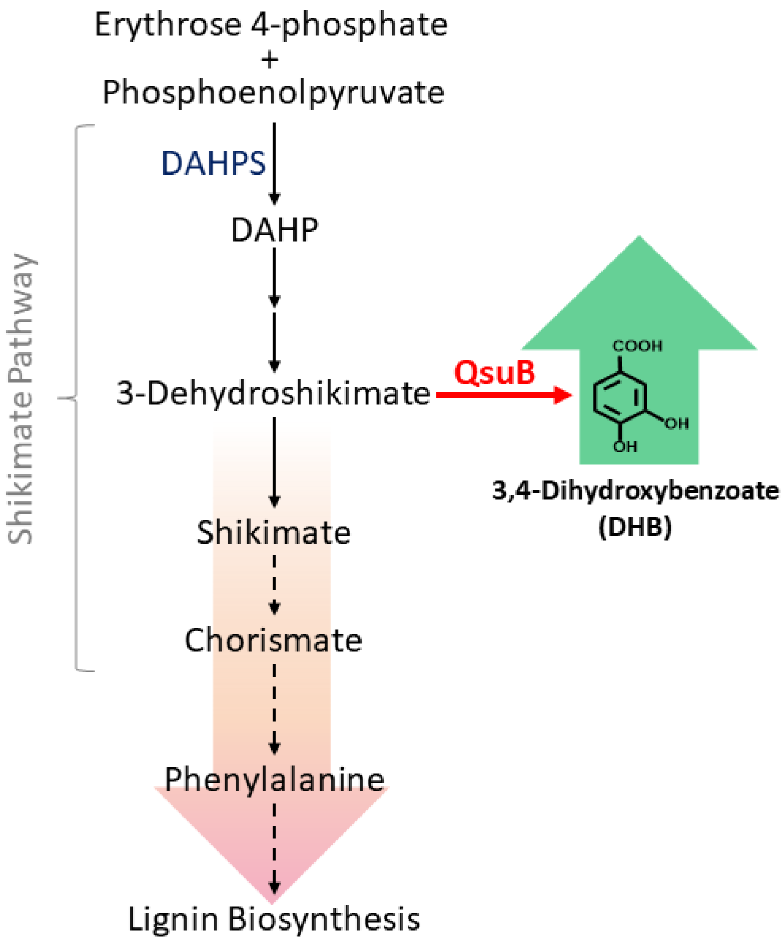

Figure S1. The QsuB engineering strategy. QsuB converts 3-dehydroshikimate into 3,4-dihydroxybenzoate (DHB) and thereby reduces the carbon flux towards shikimate and phenylalanine used for lignin biosynthesis. DAHP: 3-deoxy-D-arabinoheptulosonate 7-phosphate; DAHPS, DAHP synthase.

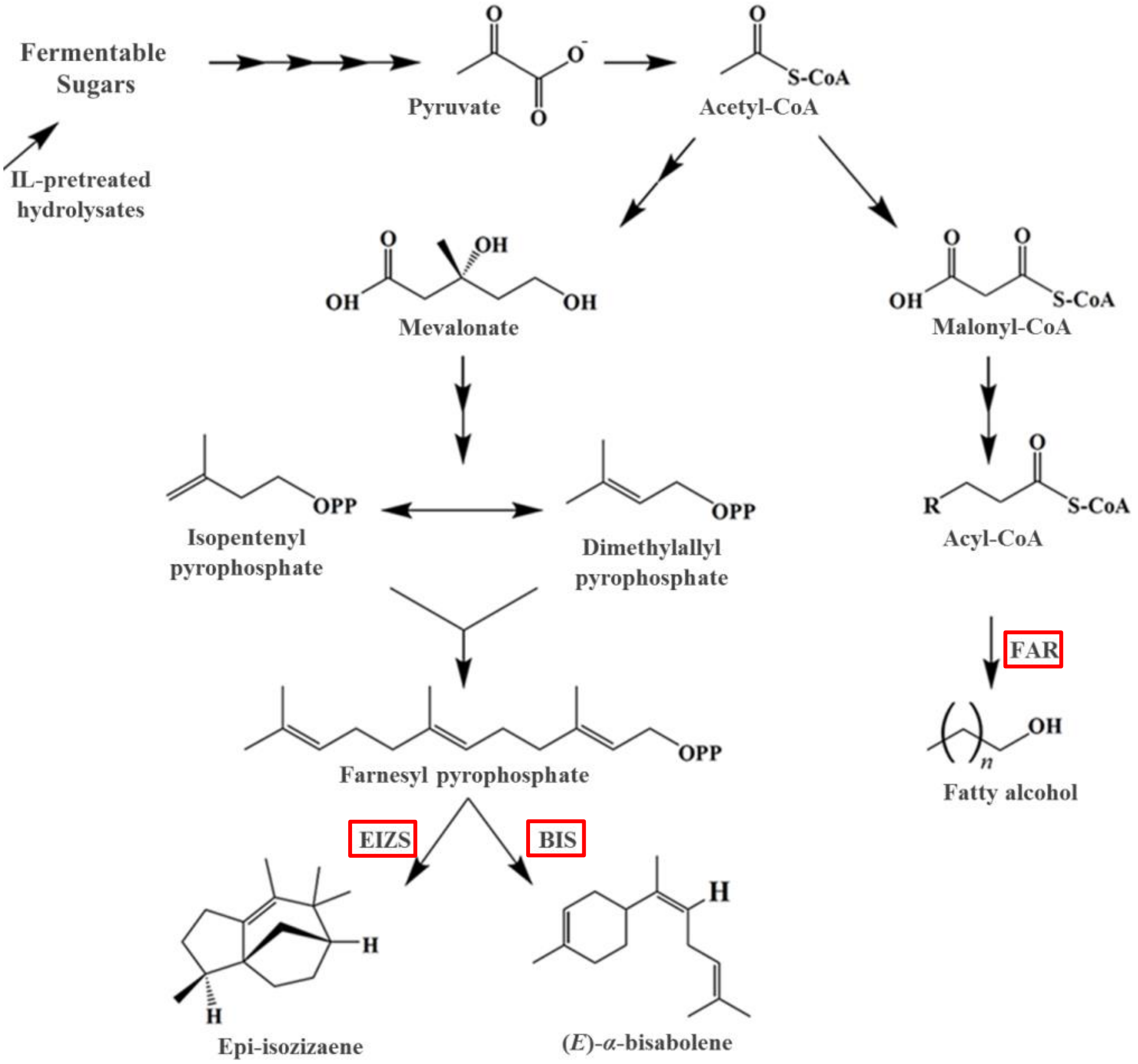

Figure S2. Metabolic routes for microbial conversion of fermentable sugars from lignocellulosic hydrolysates into sesquiterpenes (epi-isozizaene and α-bisabolene) and fatty alcohols. Engineered strains of *Rhodosporidium toruloides* containing an epi-isozizaene synthase (EIZS), an α-bisabolene synthase (BIS), or a fatty acyl-CoA reductases (FAR) are used for fermentation.

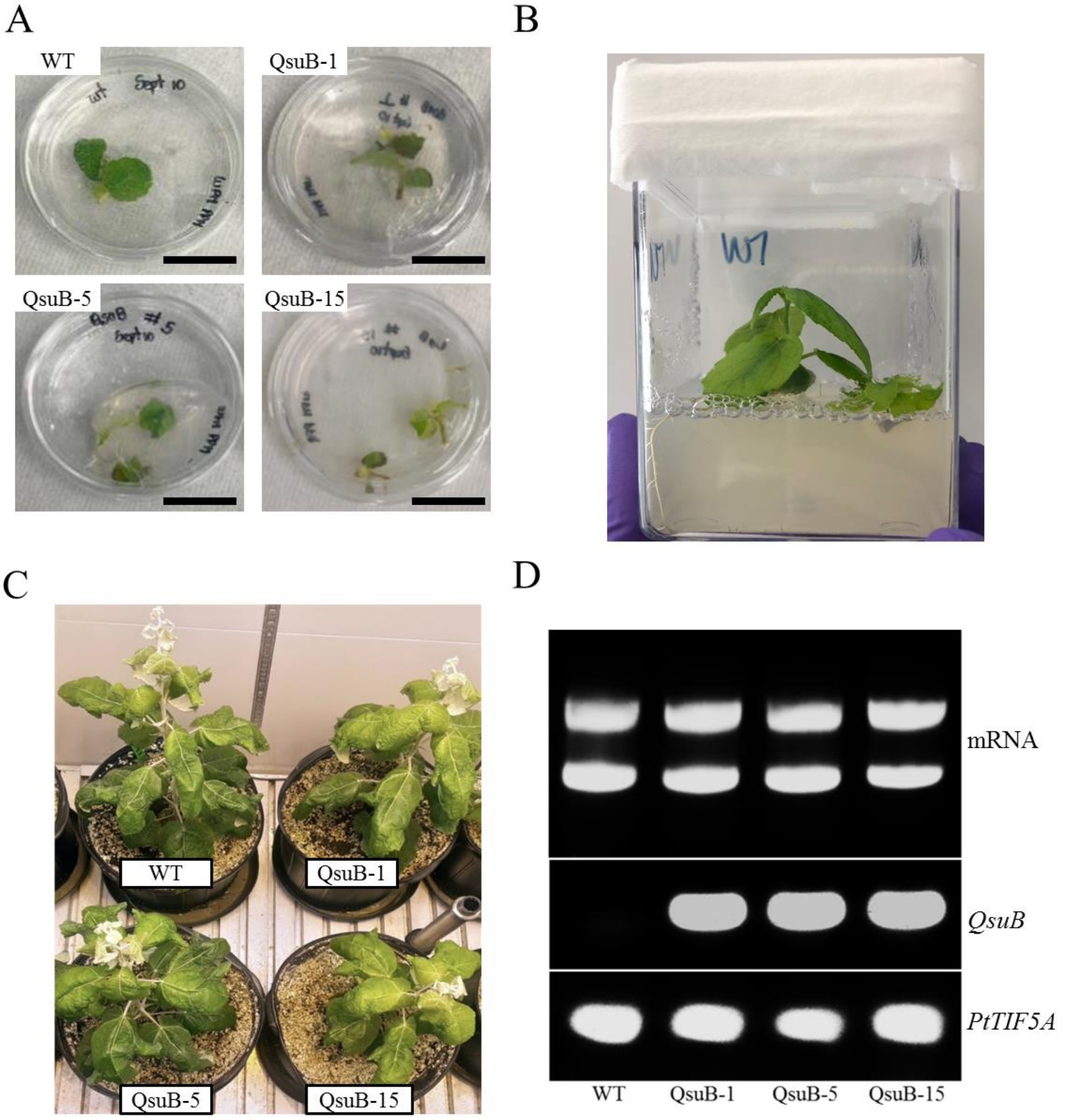

Figure S3. Propagation and characterization of WT and QsuB poplar lines. (**A**) Leaf explants (**B**) One-month-old propagated seedling with elongated roots in the rooting medium (RM). **(C)** Plants in the growth chamber one month after transfer to soil (**D**) Validation of *QsuB* expression in the stem of the QsuB transgenic lines by RT-PCR.

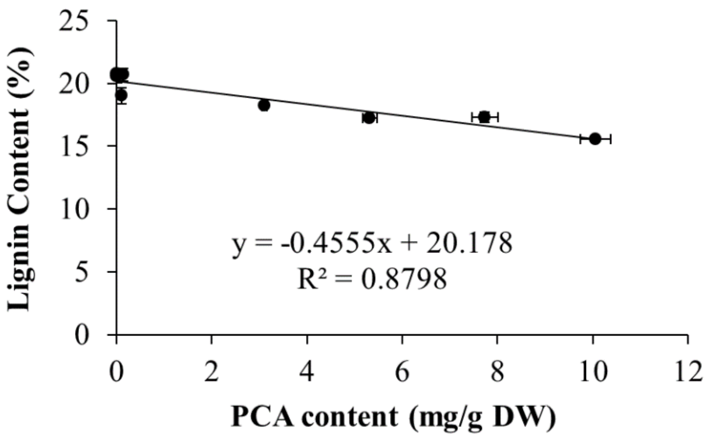

Figure S4. Correlation between total lignin content and DHB content in WT and QsuB biomass. Error bars represent the standard error of three technical replicates of the analysis of pooled biomass from eight plants.

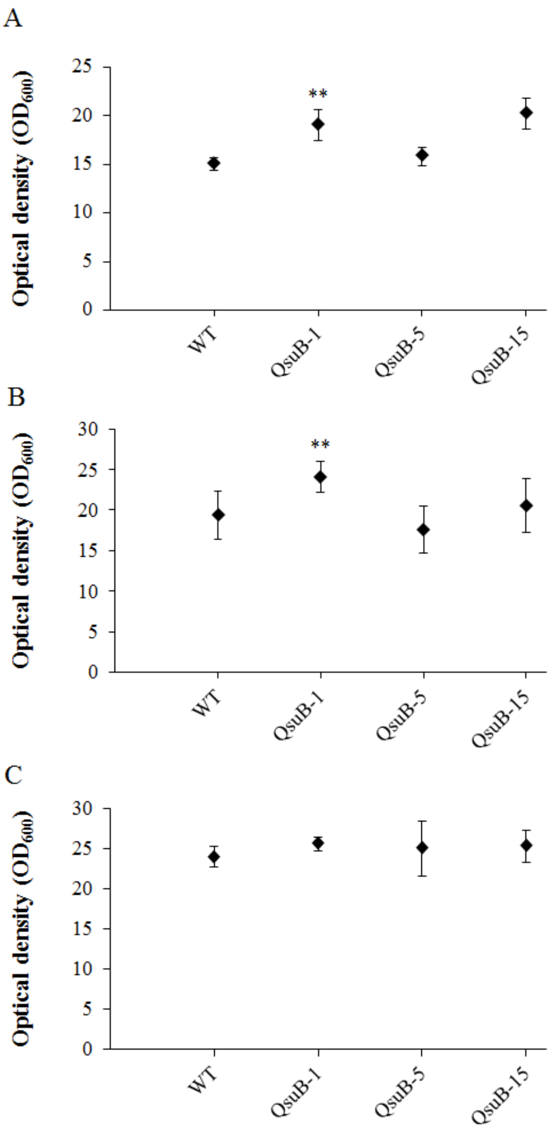

Figure S5. Optical density (OD_{600}) of *R. toruloides* cultures after cultivation on large-scale poplar hydrolysates. The strains are (**A**) GB2, (**B**) EIZS2, and (**C**) maquFOH. Error bars represent the standard error of three biological replicates from independent cultures. Asterisks indicate significant differences from the WT hydrolysate using the unpaired Student's t-test (**p* < 0.05; ***p* < 0.01).
